# Supplementary material for: Characterizing cell-type spatial relationships across length scales in spatially resolved omics data
Source: Nat Commun. 2025 Jan 3;16:350. doi: 10.1038/s41467-024-55700-1 (PMC11699133; doi:10.1038/s41467-024-55700-1)
Supplement: Supplementary file 3 — Supplementary Software 1 [file 41467_2024_55700_MOESM3_ESM.zip › CRAWDAD/plots/sim/S1_sim_trends_heatmap.pdf]

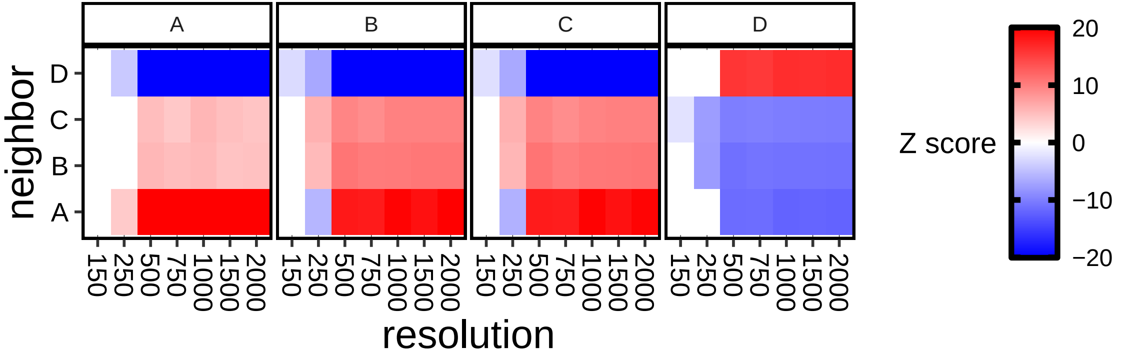

A
B
C
D
neighbor
D
C
B
A
150
250
500
750
1000
1500
2000
resolution
Z score
20
10
0
−10
−20
